# Study Crafting and Self-Undermining in Higher Education Students: A Weekly Diary Study on the Antecedents

**DOI:** 10.3390/ijerph18137090

**Published:** 2021-07-02

**Authors:** Lorena Sarah Körner, Thomas Rigotti, Kerstin Rieder

**Affiliations:** 1 Business Psychology, Aalen University, 73430 Aalen, Germany; kerstin.rieder@hs-aalen.de; 2 Work-, Organizational-, and Business Psychology, Johannes Gutenberg-University Mainz, 55122 Mainz, Germany; rigotti@uni-mainz.de; 3 Leibniz Institute for Resilience Research, 55122 Mainz, Germany

**Keywords:** study demands–resources framework, study characteristics, student engagement, student burnout, study crafting, self-undermining, higher education students

## Abstract

The aim of the current study is to validate the adaptation of the job demands–resources theory to the study context. In addition, we introduce the concepts study crafting and self-undermining to the study demands–resources framework by examining the mediating role of engagement and exhaustion in the relationship between study characteristics and study crafting and self-undermining. Over four consecutive weeks, 205 higher education students answered a questionnaire about their weekly study demands and resources, their well-being (i.e., engagement, exhaustion), and their study crafting and self-undermining behaviors. Multilevel structural equation modeling (controlling for autoregressors of mediators and dependent variables from the previous week) demonstrated a positive relationship between study resources and study crafting mediated by engagement, as well as a positive relationship between study demands and self-undermining mediated by exhaustion. Our findings show that even short-term fluctuations in study characteristics affect students’ well-being and, in turn, their proactive and dysfunctional behaviors. Accordingly, universities should provide a resource-rich study environment and limit study demands as much as possible. Furthermore, our results demonstrate that students can also actively influence their study environment themselves.

## 1. Introduction

Students are a growing part of the general population [1] and of great importance to ensuring the growth of the economy in a country [2]. At the same time, students report a high level of stress and emotional exhaustion [3]. Robins et al. [4] followed students from the final year at university through work and found that burnout was higher during study than during work. There is also a high prevalence of common mental disorders such as depression among students [2]. These findings demonstrate the importance of early interventions at universities to decrease student burnout and other stress-related psychological disorders. On the other hand, Grützmacher et al. [3] showed that approximately 50% of students hold high levels of engagement, which is especially important because evidence shows that student engagement is strongly related to student performance [5].

While there is growing interest in studying burnout and engagement among students, the majority of existing studies is instead atheoretical [6]. The job demands–resources (JD-R) model is a widespread model in the context of work that explains the development of burnout and engagement in employees [7]. Lesener et al. [8] recently introduced the study demands–resources (SD-R) framework and thus confirmed the core assumptions of the JD-R model for the higher education context. However, there is a significant gap between the findings on the JD-R model and the SD-R framework, and more research on the SD-R framework is needed [8]. Therefore, the central aim of the present study is to validate an extended version of the SD-R framework by supplementing the behavioral variables study crafting and self-undermining. These student behaviors have not yet been considered in the higher education context. However, we know from research in the work context that employees actively influence their own work, and so we assume the same is true for students. Thus, in the present study, we make a first contribution by introducing study crafting as an outcome in the SD-R framework and investigating its antecedents. We make a second contribution by introducing self-undermining as an outcome in the SD-R framework and investigating its antecedents.

Furthermore, in our study we focus on intraindividual differences by using a weekly diary approach. This approach can contribute to our understanding of student well-being and can also complement the findings of between-person studies [9]. It is often used in the work context, but there is a lack of within-person studies in a higher education context [10]. We aim to fill this research gap and make a third contribution by investigating whether the theoretical assumptions of the SD-R framework also hold when adopting a week-level within-person approach. In this way, we examined which transient study characteristics were present when students exhibited high levels of student engagement and emotional exhaustion. These characteristics, in turn, represent important starting points for fostering student engagement and proactive behavior and reducing exhaustion and dysfunctional behavior.

### 1.1. Student Burnout and Engagement

A growing number of students in Germany—almost three million in total during the winter 2020/2021 term [11]—are encountering a variety of health problems [3]. In a survey on the stress level of German students, 53% indicated a high level of stress, whereas only 5% indicated a low level of stress [12]. Approximately 25% of the students reported high levels of emotional exhaustion [3], which is the key component and most obvious manifestation of burnout [13]. Student burnout can be defined as a feeling of exhaustion caused by study demands, a detached and cynical attitude toward studying, and a feeling of incompetence as a student [5]. In our study, we focus on emotional exhaustion as a negative, as well as malleable, indicator of a student’s well-being. Emotional exhaustion can be measured on a continuum, and thus does not need to fulfill criteria for a clinical diagnosis. However, we know that emotional exhaustion is positively related to negative outcomes for the student (such as depression, fatigue, loss of motivation), as well as negative outcomes for the university (such as dropouts and lower performance) [14].

Based on a general trend toward positive psychology, which focuses on optimal human functioning rather than on malfunctioning [15], there is also a research trend toward the concept of student engagement [16]. Student engagement is characterized by a positive, fulfilling mental state that consists of vigor, dedication, and absorption. Vigor describes a high level of energy as well as the ability to invest effort. Dedication means a sense of significance and enthusiasm. Absorption is characterized by a feeling of full concentration and of being carried away. Student engagement is positively associated with performance and even more strongly linked to performance than burnout [17].

Robins et al. [6] emphasize that it is necessary to use relevant theories in the research of student burnout and engagement to obtain a stronger evidence base on their antecedents and consequences. Contrary to student well-being, employee well-being has been researched for many years, and several theoretical models are available within the work context [18]. One of the most popular models is the JD-R model [7], which was recently introduced in a higher education context by Lesener et al. [8].

### 1.2. The JD-R Theory in a Higher Education Context: The SD-R Framework

Like employees, students are involved in structured activities (e.g., attending lectures) and are aimed at specific goals (e.g., passing exams). Therefore, from a psychological perspective, there is similarity between work and study [19]. This similarity makes it possible to apply models from the work context, such as the JD-R model, to the higher education context. JD-R theory assumes that all work characteristics can be divided into two categories: job demands and job resources [20]. Accordingly, in the higher education context, we divide into two categories: study demands and study resources. Study demand*s* refer to those psychological, physical, social, or organizational study aspects that require effort and are associated with mental or physiological costs. Study resources are defined as psychological, physical, social, or organizational study aspects that contribute to goal achievement, reduce study demands, or improve personal development [7,8]. Furthermore, two psychological paths are specified within JD-R theory: the health-impairment path and the motivational path. In the health-impairment path, job demands require energy and consequently lead to exhaustion [7,21]. In the motivational path, job resources foster motivation and lead to high levels of work engagement [21].

The applicability of JD-R theory in higher education settings has been tested in initial studies [8,14,17,22]. Mokgele and Rothmann [14] first introduced the SD-R model and assumed that the paths of the JD-R model would also occur in the higher education context. The results actually showed that study demands and a lack of study resources were associated with student burnout. Conversely, study resources were associated with student engagement. As Mokgele and Rothmann [14] examined only first-year students, Gusy et al. [22] tested the paths in a sample of German students from all semesters. They confirmed a health-impairment path from study demands to reduced well-being mediated through exhaustion and a motivational path from study resources to well-being mediated through student engagement. Recently, Lesener et al. [8] introduced the SD-R framework by validating the key assumptions of the SD-R model in a large, representative sample of university students.

In the studies mentioned above, different study resources and study demands were examined that confirmed the paths specified in the SD-R framework can be initiated by different characteristics of the study. Xanthopoulou et al. [23] emphasized the importance of focusing on the characteristics that are most relevant for the studied target group in order to capture the specificity of the respective environment. Therefore, in our study, we investigated three study resources and two study demands that, on one hand, represent study characteristics as broadly as possible and, on the other hand, have been shown to be particularly important in previous studies of the SD-R framework [6,10,22]. The study resource decision latitude is known from the job demand-control model [24,25] and consists of two sub-dimensions: skill discretion and decision authority. Skill discretion means the extent to which different skills and creativity are required. Decision authority describes the autonomy to make one’s own decisions within the study program [26]. This resource captures the domain of study organization. In addition, we chose two resources that cover the interpersonal domain: social support from lecturers and social support from fellow students. Social support from lecturers refers to how attentive lecturers are and to what extent they care about their students and help them in their studies. Social support from fellow students describes interest, helpfulness, and friendliness as well as a good cooperation with fellow students [26]. Derived from the challenge–hindrance framework [27], we chose two study demands that we expected to represent a challenging and hindering demand: psychological demands and overload. Psychological demands include workload and time pressure [26]. Overload includes qualification requirements as well as qualitative overload [28]. Although an a priori categorization into challenging and hindering demands should be viewed with reservation as the interpretation is highly individual [29,30], there is empirical evidence that psychological demands are more likely to show challenge characteristics whereas overload is more likely to be perceived as a hindrance [27,31].

The vast majority of previous research on student well-being and its antecedents and outcomes is cross-sectional and uses between-person designs that focus on how students differ from one another under the assumption that the studied concepts are stable. These studies cannot explain why one individual student feels more engaged or exhausted during some weeks than they do during others because those studies ignore intraindividual variation [10]. Like Ilies et al. [9], we believe it necessary to conduct further within-person studies that extend findings from between-person studies, as both approaches complement each other. We aim to fill this gap in higher education research by investigating both paths of the SD-R framework for the first time using a weekly diary approach.

**Figure 1:**
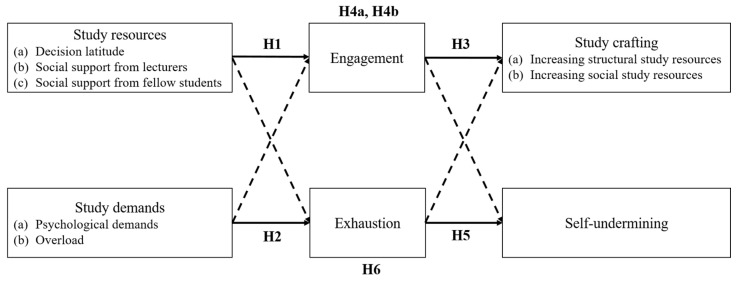
The underlying research model for the weekly relationships.

There is evidence from JD-R theory that the paths act at the between-person level but also at the within-person level and that the JD-R variables have both a stable component and a malleable component [18]. Evidence from the work context suggests that engagement and exhaustion fluctuate on a daily or weekly basis depending on existing daily or weekly demands and resources [18,32]. Bakker and Bal [33] found that teachers’ weekly job resources were positively related to their weekly work engagement. A study by Bakker et al. [10] is one of the first to examine intraindividual changes in student well-being using a weekly diary approach. Results showed that weekly study resources predicted weekly student engagement. Focusing on exhaustion, Simbula [34] showed that on days when teachers experienced higher job demands, they also reported higher daily exhaustion. It is obvious that the study program is also characterized by great variability; students have different courses and projects spread over the semester. They interact with different fellow students and lecturers, which can be rather supportive or even exhausting. In addition, exam periods are often characterized more by time pressure than is the beginning of the semester. Therefore, it is expected that study demands and study resources will fluctuate over different weeks as well. In turn, these study characteristics are expected to be relevant predictors for student engagement and student burnout in the corresponding week. Based on the theoretical and empirical findings on JD-R theory and the SD-R framework, we formulated the following hypotheses (see Figure 1):

**Hypothesis** **1** **(H1).** *There is a positive relationship between weekly study resources: (a) decision latitude, (b) social support from lecturers, and (c) social support from fellow students and weekly engagement*.

**Hypothesis** **2** **(H2).** *There is a positive relationship between weekly study demands: (a) psychological demands and (b) overload and weekly exhaustion*.

### 1.3. Study Crafting and Self-Undermining

Within JD-R theory, employees were long seen as passive, reacting to their working conditions. However, employees can also be active in modifying their own work [35,36]. Therefore, the JD-R model was extended by the bottom-up approach of job crafting. Job crafting is defined as the proactive changes employees make to their working tasks, relationships, and appraisal of their work [36], or the proactive changes of employees to their job demands and job resources [35]. These changes are aimed at better adaption of the workplace with personal skills, needs, and preferences, as well as a better fit between individual and workplace [37]. Tims et al. [37] specify four job crafting strategies: increasing structural resources, increasing social resources, increasing challenging job demands, and decreasing hindering demands. Increasing structural resources means behaviors that influence the job’s design such as changing levels of autonomy or variety within the job. Increasing social resources refers to the social aspects of the job such as searching for social support or feedback [38]. Increasing challenging job demands means behaviors such as taking on new projects. Decreasing hindering demands means, for example, the reduction of cognitive demands [37].

JD-R theory assumes that job resources foster engagement. Engaged employees want to stay engaged and therefore exhibit job crafting [20]. This process is consistent with conservation of resources (COR) theory, which states that individuals are motivated to maintain and accumulate their resources [39]. A recently published meta-analysis confirmed a reciprocal positive relationship between promotion-focused job crafting (increasing social and structural job resources, increasing challenging job demands) and engagement [40]. There is evidence that employees also craft their jobs on a daily or weekly basis [41,42,43].

We investigated the concept of study crafting in the context of higher education for the first time, as this could contribute to a more comprehensive understanding of student well-being. Because we investigated study crafting within the motivational path and therefore focused on resources, we only examined the strategies of increasing social resources and increasing structural resources. Based on the theoretical and empirical findings from the work context regarding job crafting, we made the following hypotheses (see Figure 1):

**Hypothesis** **3** **(H3).** *There is a positive relationship between weekly engagement and weekly study crafting in the form of (a) increasing structural resources and (b) increasing social resources*.

**Hypothesis** **4a** **(H4a).** *Weekly engagement mediates the relationship between weekly study resources and weekly study crafting in the form of increasing structural resources*.

**Hypothesis** **4b** **(H4b).** *Weekly engagement mediates the relationship between weekly study resources and weekly study crafting in the form of increasing social resources*.

In addition to job crafting, the behavioral component self-undermining was also included in a recent version of JD-R theory [20]. Self-undermining is defined as a “behavior that creates obstacles that may undermine performance” ([44], p. 115). Examples of self-undermining behavior are poor communication, making mistakes, and creating conflicts [45]. Within JD-R theory, it is suggested that self-undermining is the initiator of a loss cycle of strain and job demands: Employees with a high level of job demands experience higher levels of strain, which in turn promotes self-undermining [46]. Empirical research on this concept is still scarce, but an early study showed that exhaustion positively predicts self-undermining [47]. We introduced the concept to the SD-R framework, as we assumed that behaviors such as making mistakes or creating backlogs are also common among students. Derived from the JD-R theory, we made the following hypotheses (see Figure 1):

**Hypothesis** **5** **(H5).** *There is a positive relationship between weekly exhaustion and weekly self-undermining*.

**Hypothesis** **6** **(H6).** *Weekly exhaustion mediates the relationship between weekly study demands and weekly self-undermining*.

## 2. Materials and Methods

### 2.1. Participants and Procedure

Every student at Aalen University (*N* = 5573) was informed about the weekly diary study by e-mail and was given the opportunity to register. In total, 255 students signed up for the study and filled out a short demographic questionnaire upon registration. All registered students then received a web-based diary questionnaire every Friday for four consecutive weeks. Participants who completed all four weekly questionnaires had the potential to win one of five vouchers for local businesses. In addition, EUR 1 per complete diary was donated to charitable institutions. Of the registered students, 32 did not fill out any of the four questionnaires (12.5% non-responders). Participants who completed at least two of the four questionnaires were included in our analyses, resulting in a final sample of 205 participants (80.4%). Of these, 136 participants were female and 69 were male. The average age was 23 years (*SD* = 3.20, *n* = 5 missing). The students studied in the following five faculties: chemistry (*n* = 7), informatics and electronics (*n* = 22), mechanical engineering and materials technology (*n* = 27), optics and mechatronics (*n* = 35), and economics (*n* = 114). Although the number of students from the faculties varied greatly, this is still representative of Aalen University, with chemistry as the smallest faculty and economics as the largest faculty. A majority of participants (*n* = 159) studied at the bachelor’s level, and 41 studied at the master’s level (*n* = 5 missing).

### 2.2. Measures

Because participation in diary studies requires more effort from participants than filling out a one-time cross-sectional questionnaire, using shortened scales with only a few items is recommended [48]. Therefore, for each variable, we selected three items that showed the highest item total correlations in a previous cross-sectional survey as recommended by Ohly et al. [48].

*Study resources* were measured with the Questionnaire on Structural Study Conditions (StrukStud) [26], which is based on the job content questionnaire [24]. All items were adapted so that they referred to the previous week. *Decision latitude* was measured with three items. An example item is: “This week I had variety in my studies.” Cronbach’s α across the occasions ranged between 0.57 and 0.71. *Social support from lecturers/professors* (e.g., “This week my lecturers/professors helped me in my studies”; Cronbach’s α: 0.83–0.88) and *social support from fellow students* (e.g., “This week my fellow students and I worked well together”; Cronbach’s α: 0.78–0.83) were also measured with three items each. All items were answered on a 4-point Likert-type scale from “does not apply” (1) to “does apply” (4).

We investigated two *study demands*: psychological demands and overload. *Psychological demands* were measured with three items of the Questionnaire on Structural Study Conditions [26] adapted to the week level, for example: “This week, I had to work fast in my studies.” Cronbach’s α varied across the four weeks between 0.82 and 0.90. For measuring *overload,* we selected three week-level adapted items from the scale on study demands of the BARI-S/V08 [28]. An example item is, “This week my studies happened to be too difficult.” Cronbach’s α ranged from 0.73 to 0.82. All items were answered on a 4-point Likert-type scale from “does not apply” (1) to “does apply” (4).

*Study crafting* was measured with a version of the job crafting scale [38] that was adapted to the study context (Job Crafting Scale–Student Survey) [28]. The scale included three items for the sub-dimensions *increasing structural resources* (e.g., “This week I tried to learn new things during in my studies”) and *increasing social resources* (e.g., “This week I asked whether my lecturers are satisfied with my work”) that were adapted to week-level. All items were answered on a 5-point Likert-type scale from “does not apply at all” (1) to “does fully apply” (5). Cronbach’s α ranged from 0.84 to 0.89 for increasing structural resources and ranged from 0.71 to 0.84 for increasing social resources.

*Self-undermining* was measured with three items of the self-undermining scale [45]. The items were adapted to the university context and adjusted so that they referred to the previous week (e.g., “This week I created a backlog in my tasks”). The items were answered on a 7-point Likert-type scale, from “never” (1) to “always” (7). Cronbach’s α ranged between 0.69 and 0.80.

*Engagement* was measured with a 3-item version [16] of the Utrecht Work Engagement Scale–Student form (UWES-SF) [5] adapted to the week level. Each item assessed one of the three dimensions: vigor (“This week I felt strong and vigorous when I was studying”), dedication (“This week my study inspired me”), and absorption (“This week I felt happy when I was studying intensely”). The items were rated on a 7-point Likert-type scale from “never” (1) to “always” (7). Cronbach’s α for this scale across the four occasions varied between 0.81 and 0.85.

*Emotional exhaustion* was measured with the short German version (MBI-SS-KV) [49] of the Maslach Burnout Inventory–Student Survey (MBI-SS) [5]. In line with Demerouti et al. [7], only emotional exhaustion as the initial symptom of burnout was considered. The three items were adjusted so that they referred to the previous week. An example item is, “This week I felt emotionally drained by my studies.” The items were answered on a 7-point Likert-type scale from “never” (1) to “always” (7). Cronbach’s α ranged between 0.88 and 0.91.

### 2.3. Strategy of Analysis

The weekly study included four repeated measurements (Level 1; *n* = 729 occasions) nested within persons (Level 2; *N* = 205 participants). We therefore analyzed our data using multilevel path analysis in Mplus version 8.5 [50]. We employed multilevel structural equation modeling (MSEM) with concurrent models at the within and the between levels [51]. As with the person-centering approach, within effects can be interpreted as controlled for their between-level variation. Furthermore, MSEM is considered to be less prone to estimation bias [52]. In our overall model, the three study resources and two study demands were included as independent variables while the two study crafting strategies and self-undermining were included as dependent variables. Engagement and exhaustion were included as potential mediators of the relationships between study characteristics and study crafting/self-undermining. We allowed the mediators to correlate. Although we limited our hypotheses to the theoretically most relevant paths, we also included and tested additional paths in our model (dashed arrows in Figure 1).

As indirect effects typically are non-normally distributed, a distribution-free method should be used for testing mediation effects [53]. We therefore analyzed our data using the Bayesian approach [54]. Bayesian analysis provides credibility intervals (CRI) that include a predefined percentage (e.g., 95%) of posterior distribution.

Because repeated measurements data are dependent—previous states can influence current states—we corrected for serial dependency by including lagged variables [55]; we included paths from previous engagement (ratings in the previous week; lag-1) on current engagement and from previous exhaustion (ratings in the previous week; lag-1) on current exhaustion to investigate whether our independent variables predicted change in the mediators. Analogously, we included paths from previous study crafting (lag-1) on current study crafting and from previous self-undermining (lag-1) on current self-undermining to test whether our mediators predicted change in the dependent variables. Similarly, for the indirect effects we investigated whether our independent variables predicted change in the mediators (controlling for ratings of the mediator in the previous week), which in turn predicted change in the dependent variables (controlling for ratings of the dependent variables in the previous week). By including these autoregressive variables, the number of occasions at Level 1 reduced from 729 to 493.

## 3. Results

Table 1 shows means, standard deviations, intraclass correlation coefficients (ICC), and within- and between-person correlations among the study variables. The ICCs ranged between 0.33 and 0.73, confirming a sufficient within-person variation (between 26.8% for exhaustion and 66.9% for social support from lecturers), thus, justifying multilevel analysis.

### 3.1. Test of Model Fit

We conducted multilevel confirmatory factor analyses (simultaneously at the within- and between-level) to test whether the three study resources and two study demands as well as the two study crafting strategies could be discriminated. Our hypothesized model (Model 1) consisted of ten factors: three study resources (decision latitude, social support from lecturers, social support from fellow students), two study demands (psychological demands, overload), engagement, exhaustion, two study crafting strategies (increasing structural resources, increasing social resources), and self-undermining. This model yielded a good fit to the data. Still, we tested several alternative models. First, we tested Model 2, in which the three study resources were combined into one factor. Next, we tested Model 3, in which the two study demands were combined into one factor. Then we tested Model 4, in which the two study crafting strategies were combined into one factor. Finally, we tested Model 5, where the three study resources, the two study demands, and the two study crafting strategies were combined into one factor each. Table 2 shows the fit indices for the tested models, indicating best model fit for Model 1. Satorra–Bentler scaled χ*^2^* difference tests [56] also showed superior fit for our hypothesized model compared with Model 2 (*F* = 538.04, *df* = 36, *p* < 0.001), Model 3 (*F* = 425.16, *df* = 19, *p* < 0.001), Model 4 (*F* = 326.48, *df* = 19, *p* < 0.001), and Model 5 (*F* = 1424.39, *df* = 62, *p* < 0.001). We therefore conclude that our study variables show sufficient discriminant validity and included them as separate variables in our path analyses.

### 3.2. Test of Direct Effects

Regarding the relationships between the predictors and the mediator within the motivational path, we found that decision latitude (γ = 0.31, 95% Credibility interval (CRI) [0.20, 0.42]), social support from lecturers (γ = 0.17, 95% CRI [0.09, 0.26]), and social support from fellow students (γ = 0.12, 95% CRI [0.02, 0.23]) positively predicted engagement while controlling for previous engagement. Therefore, Hypothesis 1 could be confirmed. Within the health-impairment path, we found that psychological demands (γ = 0.22, 95% CRI [0.09, 0.35]) and overload (γ = 0.45, 95% CRI [0.33, 0.57]) positively predicted change in exhaustion, confirming Hypothesis 2. Results for the relationships between all predictors and mediators are shown in Table 3a.

Regarding the relationships between the mediator and the dependent variables within the motivational path, we found a positive relationship from engagement to increasing structural resources (γ = 0.25, 95% CRI [0.19, 0.31]) and from engagement to increasing social resources (γ = 0.13, 95% CRI [0.04, 0.22]), while controlling for previous study crafting. Within the health-impairment path, we found that exhaustion positively predicted change in self-undermining (γ = 0.16, 95% CRI [0.09, 0.22]). This provided full support for Hypothesis 3 and Hypothesis 5. Table 3b shows the relationships between the mediators and the outcomes. For complete reporting, the direct effects from the predictors to the outcomes are also reported in Table 3b, although no hypotheses were formulated for those relationships.

### 3.3. Test of Indirect Effects

**Figure 2:**
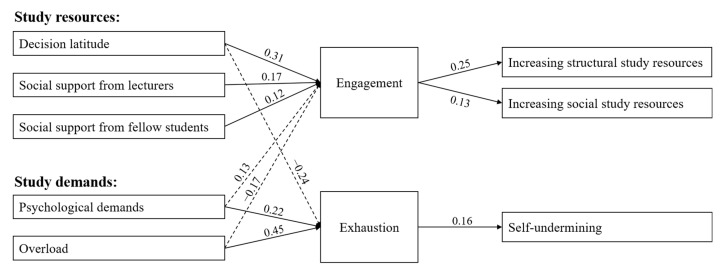
Within-person path coefficients. For ease of representation, direct effects from the predictors to the outcomes are not shown.

The indirect effects for all paths are shown in Table 4. Engagement mediated the relationship between decision latitude, social support from lecturers, as well as social support from fellow students and increasing structural resources. Engagement also mediated the relationship between decision latitude, social support from lecturers, as well as social support from fellow students and increasing social resources. Therefore, Hypothesis 4a and Hypothesis 4b were supported. Regarding the indirect effect within the health-impairment path, we found that exhaustion mediated the relationship between overload and self-undermining as well as the relationship between psychological demands and self-undermining. Therefore, Hypothesis 6 was also supported. For testing the robustness and uniqueness of our proposed indirect effects, we compared the hypothesized indirect effects with the indirect effects via the other mediator. Results showed that the indirect path from decision latitude (*p* < 0.001), social support from lecturers (*p* < 0.001), and social support from fellow students (*p* < 0.05) to increasing structural resources via engagement was significantly greater than via exhaustion. For increasing social resources, the indirect path from decision latitude (*p* < 0.01), social support from lecturers (*p* < 0.01), and social support from fellow students (*p* < 0.05) via engagement was also significantly greater than via exhaustion. On the other hand, the indirect path from psychological demands (*p* < 0.05) and overload (*p* < 0.001) to self-undermining was significantly greater via exhaustion than via engagement. This provides further support for our assumed model. Figure 2 shows the final model.

## 4. Discussion

The central aim of this study was to investigate the recently introduced SD-R framework [8] supplemented by the behavioral variables study crafting and self-undermining. More specifically, we examined whether these behaviors are also common among students and whether the underlying antecedents and processes known from the JD-R model apply in the higher education context. Hypotheses were tested using a multilevel structural equation modeling focusing on within-person effects in a weekly diary design. As expected in our hypotheses, our results showed that there was a positive relationship from study resources to study crafting, mediated by engagement within the motivational path. Within the health-impairment path, there was a positive relationship between study demands and self-undermining, mediated by exhaustion. Our study confirms that study characteristics fluctuate over time and that these variations affect student well-being as well as their proactive and dysfunctional behavior.

Previous cross-sectional research has found positive relationships between study resources and engagement and between study demands and exhaustion at the between-person level [8,14,22]. Our results indicate that, regardless of the general availability of study resources, even a weekly (and therefore short-term) experience of decision latitude or social support is relevant for fostering engagement. On the other hand, short-term experience of overload or psychological demands increases exhaustion, regardless of the general level of these study demands. Thus, we extend previous findings by confirming both paths of the SD-R framework at the within-person level for the first time. The results of our study correspond with findings from other studies from the work context, where positive relationships from job resources to engagement and from job demands to exhaustion were already found on a daily basis [23,34,57] and a weekly basis [33].

Looking at study crafting, we found that engagement positively predicted increasing structural and social resources. Furthermore, engagement mediated the relationships between all investigated study resources and the two study crafting strategies. Within the health-impairment path, we found that exhaustion positively predicted self-undermining and mediated the relationships between all investigated study demands and self-undermining. Previous studies on the SD-R framework examined the mediating role of engagement and exhaustion in the relationship between study characteristics and other outcomes such as health [8,22], satisfaction with life [14], and performance [17]. Our findings on the outcomes study crafting and self-undermining are unique in the higher education context as, to the best of our knowledge, this is the first time these variables have been studied. Consequently, no comparisons of our study with research in this context is possible. However, our findings are in line with the theoretical assumptions of JD-R theory that suggest that, within the motivational path, job resources foster engagement and engaged employees, in turn, show job crafting behavior [20]. Within the health-impairment path, it is assumed that job demands are positively related to self-undermining through strain [46]. Consistent with our findings, there is also empirical evidence from the work context that engagement positively predicts job crafting [58,59,60,61] and that exhaustion positively predicts self-undermining [47].

Our additional analyses further revealed that in addition to the indirect effects, there were also direct effects from study resources on study crafting. This corresponds with conservation of resources (COR) theory, which states that people try to conserve but also accumulate their resources [39]. There were also direct effects from study demands to study crafting. Again, this is consistent with COR theory, as it is assumed that resource gain cycles are most likely to occur in highly stressful situations [62]. Consequently, people should strive to increase their resources (e.g., through study crafting) when there are many demands. There is also empirical evidence for this assumption from a daily diary study in the work context. Petrou et al. [63] showed that employees engaged in higher levels of seeking resources on days that they experienced high job resources and high job demands.

Regarding the individual study characteristics, the strongest effect on engagement and the strongest indirect effect on study crafting through engagement were found for decision latitude. There is evidence that this study resource is associated with other positive outcomes such as study satisfaction [26,64,65] or well-being [66], which underscores the importance of this resource. Our additional analyses further revealed a significant negative relationship from decision latitude to exhaustion, which is also in line with previous findings that confirmed a negative effect from study resources on burnout [8].

Within the health-impairment path, the direct effect on exhaustion and the indirect effect on self-undermining through exhaustion was stronger for overload than for psychological demands. Additional analyses further revealed a significant positive relationship from psychological demands to engagement and a significant negative relationship from overload to engagement. This can be explained using the challenge–hindrance framework [27]. This framework builds on the transactional stress model, which assumes that people evaluate stressors as hindrances or challenges depending on their personal coping strategies [67,68]. Studies from the higher education context confirm that hindrance stress has a negative effect on motivation to learn whereas challenge stress has a positive effect on motivation to learn; however, both forms show a positive relationship with exhaustion [27]. As assumed, based on our results, we conclude that overload is interpreted as a hindrance stressor, whereas most of the participants in our study interpreted psychological demands as a challenge stressor.

### 4.1. Theoretical Contributions

Our study adds to the SD-R framework [8] in three ways. First, we were able to show that the motivational path can be extended to include the variable study crafting. We define study crafting—in analogy to job crafting—as the proactive changes that students make in their study demands and study resources, and therefore the active influence of the student on his or her study environment [35,36]. This change is aimed at a better adaption of the study environment with personal skills, needs, and preferences and therefore a better fit between student and study [37]. With our findings, we also contribute to the job crafting literature, as this concept is already researched in a variety of domains such as leisure crafting [69], home crafting [70], and work–life balance crafting [71]. We confirm that higher education is also a context in which crafting behavior is exhibited. Second, regarding the health-impairment path, we were able to show that it can be extended to include self-undermining. Derived from the work context, we define self-undermining in the study context as a behavior creating obstacles that may in turn lower academic performance [44,45]. Finally, the present study makes a third contribution by providing insights into the dynamic processes of the SD-R framework through the application of a person-centered approach. We extend findings from previous cross-sectional studies on the SD-R framework by confirming that the paths not only act at a general level, but also at a weekly within-person level. Considering the concepts as transient states enlarges our understanding of how student well-being is affected over short periods of time.

### 4.2. Limitations and Suggestions for Future Research

Our study is not without limitations, but it also offers many starting points for future research. First, all data were collected using self-report measures, which raises concerns about common-method bias [72]. However, Spector [73] states that these concerns are often overstated. Because diary research focuses on within-person variation, the influence of response tendencies, such as self-serving bias between persons, is removed. Nevertheless, future studies could combine self-rated measures with other-rated measures (e.g., fellow students, lecturers) and integrate objective measures such as performance (GPA).

Investigating intraindividual effects represents a strength of our study, as we were able to show that the examined paths act at a weekly level. Because we included autoregressors, we can further confirm the robustness of our results. Thus, within-person research seems promising also in the context of higher education. At the same time, we could not ultimately test the directionality of our hypothesized paths with our study. Longitudinal studies could examine causal relationships. As JD-R theory assumes that the variables are dynamically interrelated and continuously influence each other in the form of a gain cycle and a loss cycle, it should be investigated whether these reciprocal relationships also occur within the SD-R framework [20].

Regarding our sample, another limitation is the relatively small number of study participants. However, this is typical for diary studies because participation here requires a comparatively high effort [48]. Furthermore, all students voluntarily participated in our study, which may have caused a self-selection bias. Further studies are warranted to replicate and refine the presented empirical results.

Although we already examined a variety of study resources and study demands, further research could benefit from testing additional resources and demands to obtain a more comprehensive understanding of which short-time study characteristics are most important for student well-being and proactive behavior. In addition, a further step could be to examine personal resources and personal demands because initial studies show that these can also influence student engagement [10].

As we investigated study crafting and self-undermining in the higher education context for the first time, validation of the scales for assessing these constructs is still pending. Bindl et al. [74] recently introduced a new framework to assess job crafting that distinguishes eight job crafting strategies. It would be worth examining which of these strategies are relevant for students in future studies to obtain a better understanding of crafting in the context of higher education.

A final limitation is that data collection took place during the COVID-19 pandemic and thus during a time that was predominantly characterized by online lectures. This limits the ability to generalize the results to a certain extent, which is why further investigations are necessary when face-to-face study begins again.

### 4.3. Practical Implications

A significant number of students suffer from stress, exhaustion, and mental disorders [2,3]. At the same time, many students hold high levels of engagement, which is positively associated with, for example, performance, satisfaction with life, and health [5,14,22]. The results of our study demonstrate the crucial role of study characteristics in fostering engagement on one hand and preventing exhaustion on the other, especially because variation in engagement and exhaustion influences proactive and dysfunctional student behavior. Therefore, a practical implication of our study for universities is to take care to provide or encourage a resourceful study environment. Academic staff, professors, and lecturers should take care to design study programs in such a way that students have sufficient decision latitude, and they should support students in the best possible way. Social support among students should also be encouraged; for example, by forming learning groups in courses. On the other hand, care should be taken to limit study demands as much as possible, in particular those that the majority of students consider to be hindrances, such as unclear expectations or stalled learning progression [27]. Although challenging demands may foster engagement, we do not conclude that they are beneficial, per se, as they also show a strong relation with exhaustion. Furthermore, there is evidence from the work context that engagement benefits from day-specific challenging demands only on days when a high level of job resources is available [57]. Because our study showed that study characteristics fluctuate on a weekly basis and thus in the short term, attention should be paid to sufficient resources especially during stressful periods; for example, during examination phases. This may help counteract exhaustion and self-undermining and, in contrast, to promote engagement and study crafting.

We assume that there is no optimal level of study resources and study demands that fits all students. In our study, we were able to show that students—just like employees—are not just passive recipients in their studies, but can become active and adapt their study environment themselves via study crafting. For this reason, we see a second practical implication in relying on bottom-up approaches aimed at optimizing the study environment at the individual level [75]. In the work context, a recent review confirms the beneficial effects of job crafting interventions on motivation, well-being, performance, and strain reduction [76]. Therefore, the next step should be to establish study crafting interventions in which students learn to self-adjust their study demands and study resources and therefore optimize the fit between study and student. Because we assume that behaviors established during studies persist into working life, these interventions can also function as a primary intervention with regard to health in the work context.

## 5. Conclusions

As student well-being is of growing interest, a theoretical basis for examining the underlying processes is of great importance. The SD-R framework provides an initial foundation in this regard. Our study adds to existing knowledge of this framework by replicating the findings from between-person studies at the within-person level. Thus, it becomes clear that the theoretical assumptions of the SD-R framework also hold at the weekly level and that dynamic processes should also be included. Furthermore, we expanded the motivational path and the health-impairment path by introducing the concepts of study crafting and self-undermining in the higher education context for the first time. In line with our hypotheses, we confirm that there is a positive relationship between study resources and engagement and between engagement and study crafting and that engagement mediates the relationship between study resources and study crafting within the motivational path. Within the health-impairment path, we confirm that there is a positive relationship between study demands and exhaustion and between exhaustion and self-undermining and that exhaustion mediates the relationship between study demands and self-undermining. Our study hereby helps to narrow the knowledge gap between the widely researched JD-R model and the little-researched SD-R framework. As we are still at the very beginning of research on study crafting and self-undermining in the higher education context, we hope that our study will encourage further research in this area.

## Figures and Tables

**Table 1:** Means, standard deviations, ICCs, and intercorrelations of all study variables.

Variable	M	SD	ICC	1	2	3	4	5	6	7	8	9	10
1. Decision latitude	2.50	0.64	0.41		0.30 ***	0.16 **	0.09	−0.09 *	0.31 ***	−0.16 **	0.29 ***	0.19 ***	−0.10
2. Support lecturers	2.68	0.74	0.33	0.57 ***		0.12 **	0.13 ***	−0.05	0.26 ***	−0.04	0.22 ***	0.21 ***	0.03
3. Support fellow students	3.26	0.67	0.50	0.21	0.41 ***		0.14 **	0.06	0.16 ***	−0.02	0.13 **	0.06	0.03
4. Psychological demands	3.11	0.79	0.56	−0.03	−0.07	−0.06		0.28 ***	0.12 *	0.20 ***	0.32 ***	0.12 **	0.34 ***
5. Overload	2.52	0.78	0.55	−0.18 *	−0.11	−0.03	0.69 ***		0.13 *	0.33 ***	0.14**	0.14**	0.40 ***
6. Engagement	3.25	1.18	0.62	0.58 ***	0.28 **	−0.05	−0.22 **	−0.41 ***		−0.33 ***	0.39 ***	0.17 ***	−0.05
7. Exhaustion	3.98	1.72	0.73	−0.32 ***	−0.20 *	−0.14	0.72 ***	0.74 ***	−0.57 ***		−0.06	0.02	0.32 ***
8. Increasing str. resources	3.77	0.84	0.45	0.55 ***	0.28 **	0.13	0.25 **	0.11	0.42 ***	−0.01		0.20 ***	0.07
9. Increasing soc. resources	2.21	1.07	0.48	0.43 ***	0.32 ***	0.01	0.16	0.11	0.28 **	0.09	0.40 ***		0.04
10. Self-undermining	3.60	1.38	0.67	−0.14	−0.13	−0.10	0.60 ***	0.70 ***	−0.34 ***	0.70 ***	0.06	0.10	

Note: *N* = 205. Number of observations = 729. Within-person correlations are above the diagonal and between-person correlations are below the diagonal. ICC = intraclass correlation coefficients. * *p* ≤ 0.05. ** *p* ≤ 0.01. *** *p* ≤ 0.001.

**Table 2:** Model fit indices for the tested models.

Fit indices	Model 1	Model 2	Model 3	Model 4	Model 5
	10 Factors	8 Factors (Three Study Resources Combined into One Factor)	9 Factors (Two Study Demands Combined into One Factor)	9 Factors (Two Study Crafting Strategies Combined into One Factor)	6 Factors (Three Study Resources, Two Study Demands, Two Study Crafting Strategies Combined into One Factor Each)
χ*^2^*(*df*)	1210.75 (721)	1820.45 (757)	1611.20 (740)	1570.01 (740)	2621.59 (738)
AIC	53,140.38	53,704.36	53,633.96	53,473.52	54,453.31
BIC	54,237.79	54,636.47	54,575.25	54,483.69	55,266.04
CFI	0.94	0.86	0.89	0.89	0.76
TLI	0.92	0.84	0.87	0.88	0.74
RMSEA	0.03	0.04	0.04	0.04	0.06
SRMR (within)	0.05	0.07	0.07	0.08	0.11
SRMR (between)	0.07	0.17	0.09	0.12	0.21

Note: *df* = degrees of freedom; AIC = Akaike information criterion; BIC = Bayesian information criterion, CFI = comparative fit index; TLI = Tucker–Lewis index; RMSEA = root mean square error of approximation; SRMR = standardized root mean square residual.

**Table 3:** Within-person path coefficients and credibility intervals for the mediators and the outcomes.

**(a) Results for the Mediators**						
**Variable**	**Engagement**		**Exhaustion**			
	**γ**	**95% CRI**	**γ**		**95% CRI**	
Decision latitude	**0.31**	**[0.20, 0.42]**	**−0.24**		**[−0.37, −0.11]**	
Social support from lecturers	**0.17**	**[0.09, 0.26]**	0.03		[−0.08, 0.13]	
Social support from fellow students	**0.12**	**[0.02, 0.23]**	−0.07		[−0.19, 0.06]	
Psychological demands	**0.13**	**[0.02, 0.23]**	**0.22**		**[0.09, 0.35]**	
Overload	**−0.17**	**[−0.27, −0.07]**	**0.45**		**[0.33, 0.57]**	
Engagement (lag-1)	**−0.21**	**[−0.30, −0.12]**				
Exhaustion (lag-1)			**−0.24**		**[−0.33, −0.16]**	
**(b) Results for the Outcomes**						
**Variable**	**Increasing Structural Resources**		**Increasing Social Resources**		**Self-Undermining**	
	**γ**	**95% CRI**	**γ**	**95% CRI**	**γ**	**95% CRI**
Engagement	**0.25**	**[0.19, 0.31]**	**0.13**	**[0.04, 0.22]**	0.04	[−0.04, 0.12]
Exhaustion	−0.01	[−0.06, 0.04]	0.03	[−0.04, 0.10]	**0.16**	**[0.09, 0.22]**
Decision latitude	**0.19**	**[0.10, 0.28]**	**0.17**	**[0.05, 0.30]**	**−0.12**	**[−0.23, −0.01]**
Social support from lecturers	0.06	[−0.01, 0.12]	**0.18**	**[0.08, 0.28]**	0.06	[−0.03, 0.15]
Social support from fellow students	0.01	[−0.08, 0.09]	−0.02	[−0.14, 0.10]	−0.00	[−0.11, 0.11]
Psychological demands	**0.25**	**[0.16, 0.33]**	0.04	[−0.07, 0.16]	**0.28**	**[0.17, 0.39]**
Overload	**0.14**	**[0.06, 0.22]**	**0.21**	**[0.09, 0.32]**	**0.39**	**[0.29, 0.50]**
Increasing structural resources (lag-1)	**−0.15**	**[−0.23, −0.06]**				
Increasing social resources (lag-1)			**−0.13**	**[−0.22, −0.04]**		
Self-undermining (lag-1)					**−0.22**	**[−0.31, −0.13]**

Note: γ = unstandardized path coefficient. CRI = credibility interval. Credibility intervals that do not contain 0 are in bold style.

**Table 4:** Within-person path coefficients and credibility intervals for the indirect effects.

Predictor	Mediator	Outcome	γ	95% CRI
Hypothesized indirect effects				
Decision latitude	Engagement	Increasing structural resources	**0.08**	**[0.05, 0.11]**
Social support lecturers	Engagement	Increasing structural resources	**0.04**	**[0.02, 0.07]**
Social support fellow students	Engagement	Increasing structural resources	**0.03**	**[0.00, 0.06]**
Decision latitude	Engagement	Increasing social resources	**0.04**	**[0.01, 0.08]**
Social support lecturers	Engagement	Increasing social resources	**0.02**	**[0.01, 0.05]**
Social support fellow students	Engagement	Increasing social resources	**0.01**	**[0.00, 0.04]**
Psychological demands	Exhaustion	Self-undermining	**0.03**	**[0.01, 0.06]**
Overload	Exhaustion	Self-undermining	**0.07**	**[0.04, 0.11]**
Additional indirect effects				
Psychological demands	Engagement	Increasing structural resources	**0.03**	**[0.01, 0.06]**
Overload	Engagement	Increasing structural resources	**−0.04**	**[−0.07, −0.02]**
Psychological demands	Engagement	Increasing social resources	**0.02**	**[0.00, 0.04]**
Overload	Engagement	Increasing social resources	**−0.02**	**[−0.05, −0.01]**
Decision latitude	Engagement	Self-undermining	0.01	[−0.01, 0.04]
Social support lecturers	Engagement	Self-undermining	0.01	[−0.01, 0.02]
Social support fellow students	Engagement	Self-undermining	0.00	[−0.01, 0.02]
Psychological demands	Engagement	Self-undermining	0.00	[−0.01, 0.02]
Overload	Engagement	Self-undermining	−0.01	[−0.02, 0.01]
Decision latitude	Exhaustion	Increasing structural resources	0.00	[−0.01, 0.02]
Social support lecturers	Exhaustion	Increasing structural resources	0.00	[−0.00, 0.00]
Social support fellow students	Exhaustion	Increasing structural resources	0.00	[−0.00, 0.01]
Psychological demands	Exhaustion	Increasing structural resources	−0.00	[−0.01, 0.01]
Overload	Exhaustion	Increasing structural resources	−0.00	[−0.03, 0.02]
Decision latitude	Exhaustion	Increasing social resources	−0.01	[−0.03, 0.01]
Social support lecturers	Exhaustion	Increasing social resources	0.00	[−0.00, 0.01]
Social support fellow students	Exhaustion	Increasing social resources	−0.00	[−0.01, 0.01]
Psychological demands	Exhaustion	Increasing social resources	0.01	[−0.01, 0.02]
Overload	Exhaustion	Increasing social resources	0.01	[−0.02, 0.05]
Decision latitude	Exhaustion	Self-undermining	**−0.04**	**[−0.07, −0.01]**
Social support lecturers	Exhaustion	Self-undermining	0.00	[−0.01, 0.02]
Social support fellow students	Exhaustion	Self-undermining	−0.01	[−0.03, 0.01]

Note: γ = unstandardized path coefficient. CRI = credibility interval. Credibility intervals that do not contain 0 are in bold style.

## Data Availability

Data used in this paper will be made available by the corresponding author upon request.
